# Potential research participants’ use of information during the consent process: A qualitative pilot study of patients enrolled in a clinical trial

**DOI:** 10.1371/journal.pone.0234388

**Published:** 2020-06-18

**Authors:** Simon Paul Jenkins, Melanie J. Calvert, Heather Draper

**Affiliations:** 1 Division of Health Sciences, Warwick Medical School, University of Warwick, Coventry, West Midlands, United Kingdom; 2 Centre for Patient Reported Outcomes Research, Institute of Applied Health Research, University of Birmingham, Birmingham, West Midlands, United Kingdom; 3 National Institute for Health Research Birmingham Biomedical Research Centre, University of Birmingham, Birmingham, West Midlands, United Kingdom; 4 National Institute for Health Research Applied Research Centre West Midlands, and National Institute for Health Research Surgical Reconstruction and Microbiology Research Centre, University of Birmingham, Birmingham, West Midlands, United Kingdom; 5 Birmingham Health Partners Centre for Regulatory Science and Innovation, Birmingham, West Midlands, United Kingdom; Universiteit van Amsterdam, NETHERLANDS

## Abstract

There is increasing evidence that clinical trial participants are uninformed about the trials in which they participate, raising ethical concerns regarding informed consent. The aim of this pilot study was to explore clinical trial participants’ use of consent discussions and information sheets when considering participating in clinical trials research. A qualitative, interview-based pilot study was designed in order to elicit, through dialogue, details of the reasons for participants’ use of, and preferences regarding, different modes of information provision. Semi-structured interviews were undertaken with two different groups of patients who were participants in the Reinforcement of Closure of Stoma Site trial. The first group comprised newly-consented trial participants, who had been recruited up to 72 hours before our interview; the second group comprised patients attending a follow-up clinic 12 months after joining the trial. Thirteen participants were recruited in total: three newly-consented patients, and ten follow-up patients. The study found that participants’ use of consent discussions to gain information about clinical trials was varied, and that they only minimally used information sheets after providing initial consent for the trial. Participants demonstrated varying degrees of knowledge about the trial, with some having forgotten that they were still involved in the trial. Participants reported a high level of trust in medical staff as a reason for not seeking more information about the trial. Some participants reported dissatisfaction with the timing of information provision. Some were amenable to novel ways of receiving trial information, such as web-based methods. The pilot study demonstrated the feasibility of a larger study into the provision of information to prospective clinical trial participants. The results suggest that considering alternative ways of providing information and the appropriateness of existing information provision may be acceptable to and useful for potential trial participants.

## Introduction

Clinical trials provide valuable evidence on the efficacy and effectiveness of healthcare interventions and this evidence can inform clinical practice and health policy. Gaining properly *informed* consent to participation in research is said to demonstrate respect for research participants and for their autonomy [1–4]. While theoretical and practical objections to the use of informed consent to preserve autonomy have been raised (for example, it cannot be obtained from those without capacity to consent, and produces quandaries when applied to the vulnerable or dependent, whose consent may not be valid if they feel coerced [5]), the process nevertheless remains a legal prerequisite for the inclusion of humans as participants in most forms of research. The Nuremberg Code emphasised participants’ ‘sufficient knowledge and comprehension of the elements of the subject matter involved’ as a necessary condition for consent [6]. More recently, the importance of information in consent has been codified in the Declaration of Helsinki [7], the EU Clinical Trials Directive (2001/20/EC) [8], and in the UK, the Medicines for Human Use (Clinical Trials) Regulation 2004 [9].

Participant information sheets (PIS) are routinely provided as a basis for discussion between the potential research participant and the healthcare/research team about taking part. PIS are, however, becoming increasingly long, which may deter some potential research participants, thereby limiting the generalizability of research results. Furthermore, previous research has shown that some participants are willing to participate in research despite understanding little or none of the information provided [10,11]. It is therefore unsurprising that analysis of the consent process for research has highlighted many studies whose participants had incomplete or inaccurate understanding of the trials in which they were participating [12–17]. Studies have concluded that research participants’ recall of information about the trial on which they are enrolled is low, and they are often unaware of the aim of the trial and the fact that their participation is voluntary [18,19].

In this paper, we report the results of pilot work aimed at determining: 1) the extent to which potential participants, at the time of giving consent, rely on information sheets and/or consent discussions to inform their decision to participate; 2) the extent to which participants consult their information sheet at other points during a trial, and for what purposes. This pilot study adds to the existing body of work exploring participants’ experience of the consent process and their use of opportunities to get trial information. In particular, this study focused on participants’ use of consent discussions and written information resources throughout the process of being trial participants. This study is a precursor to future work that will investigate better ways of providing information to participants.

## Methods

A qualitative pilot study was designed to explore whether and to what extent participants used written information and/or consent discussions to inform their decisions. This was intended to determine the feasibility of a larger study looking at ways to improve the quality of the information-sharing part of the consent process. By “consent discussion” we mean the meeting during which the research participant discussed the research with a member of the research team, had an opportunity to ask questions, decided to take part, and recorded formal consent, usually in writing, and witnessed by the team member [20]. According to Good Clinical Practice (GCP) guidelines, the consent discussion should recap the main points in the information sheet, which is typically provided ahead of time. If potential participants are not reading the information sheets before the consent discussion, the interview becomes a crucial means by which information is provided prior to securing consent to participate. A qualitative, interview-based method was appropriate to meet the aims of the study, given that we hoped to elicit, through dialogue, details of the reasons and rationales behind participants’ use of, and preferences regarding, different modes of information provision.

The PIS is intended not just to inform a consent decision, it is also a point of reference for information that a participant might want during a trial. To understand how the PIS was being used in both contexts, we wanted to interview people who had recently given consent to participate and those who had been in a trial for several months.

Clinical trials units at the University of Birmingham were contacted to identify potential trials in which this qualitative study could be embedded. Our inclusion and exclusion criteria, based on our aims, were as follows:

### Inclusion criteria–stage one

Anyone over the age of 18, with capacity to consent, who had agreed to participate in a clinical trial within 72 hours prior to the interview.

### Inclusion criteria–stage two

Anyone over the age of 18, with capacity to consent, who was currently participating in a trial and had been enrolled on that trial for at least a month at the time of interview.

### Exclusion criteria–both stages

Anyone unwilling to participate.

Anyone who lacked capacity to consent

### Sampling

Sampling of study participants was constrained by the need to identify an ongoing clinical trial being conducted by researchers who were: willing to allow us to embed our study into their trial, including seeking an amendment to their research ethics approval, and speak to their participants; recruiting participants at a sufficient rate for us to gather our data within the 6-month study period; recruiting participants in such a way that it would not be unduly burdensome to recruit them to a further study shortly afterwards.

The Reinforcement of Closure of Stoma Site (ROCSS) trial [21] met these criteria. Patients undergoing stoma reversal surgery and who agreed to participant in ROCSS were randomised either to receive a biological mesh to strengthen the stoma site or to receive the standard available therapy (i.e. no such mesh). Participation in this trial was not regarded as posing a high risk; participants were undergoing surgery anyway. Nevertheless, the inclusion of the mesh intervention was not entirely risk free, so a reasonably large amount of information provision for participants might be expected.

Convenience sampling was used to select participants in the ROCSS trial at one of its sites. Participants were approached at their regular clinical appointments by a member of the ROCSS team, and asked for their permission for a researcher to speak to them. No record was kept of participants who refused this permission. ROCSS participants who expressed an interest in participating in our study were met individually by SJ. SJ explained the pilot study information sheet to them, and those who gave consent recorded this on a consent form.

### Ethics approval and consent to participate

Our pilot study protocol, information sheets, and consent forms were submitted as an amendment to the ROCSS trial (favourable opinion reference 12/WM/0187 –Coventry and Warwickshire Research Ethics Committee). Appropriate local Research and Development (R&D) permission was gained to access participants at the Queen Elizabeth Hospital, Birmingham. All interviewees gave written consent to participate.

### Interviews

#### Research team and reflexivity

SJ conducted the interviews from May to July 2014, in a private room in the hospital where the participant was having their clinical visit. There was no relationship between the research team and the participants prior to commencing the study, and participants were told that the interviewer was a researcher from a nearby university. HD and MC had already published work on problems with the informed consent process [11,15], so confirmation bias may have influenced their interpretation of the data.

#### Study design

The interviews were semi-structured, making use of a topic guide (S1 Appendix) with various prompts [22], which ensured that data was gathered consistently. Questions on the topic guide asked about: which part(s) of the research the participants found striking or influenced their decision whether to participate; what the positives and negatives about participating would be; which questions they asked in the consent discussions; where they kept and how much they referred back to written information (the information sheet) about the trial; what kind of information they would refer back to and what kind of information they thought others might refer back to in the information sheet; and, what they thought about different modes of information provision, including an electronic expanding information sheet. The questions were open-ended, however, so that participants had the opportunity to raise and discuss issues that were important or interesting to them [23]. Part of the strategy for determining participants’ use of information was to attempt to establish how much they knew about the trial in which they were participating. Rather than gathering the data through the use of direct testing of knowledge, questions were posed in an indirect way, in order to mitigate the risk of participants feeling “tested” on their understanding or embarrassed if they were unable to provide answers. This ethical decision led us to design a topic guide with open questions, bringing our interview approach slightly further towards a narrative interview [24,25], as we asked participants to provide us with a broader account of their experience of the consent process.

### Analysis

Data from the interviews were transcribed verbatim from the audio recordings. SJ read the transcripts several times and then coded them for relevant themes. Coding was undertaken by a single researcher (SJ). HD read all of the interview transcripts, and themes were reviewed by experts in ethics and trials (HD and MC). The analysis followed a process of iterative content analysis, using constant comparison methods [26], where codes were refined and grouped into categories as the coding process progressed. Cases that were inductively inconsistent with the emerging themes were identified and given special consideration, in line with the process of deviant case analysis outlined by Silverman [27]. Meetings were held between the authors to discuss and review the identified themes.

## Results

Thirteen participants were recruited to our study from the ROCCS trial (Table 1).

**Table 1:** Data collection phases and participants.

Stage	Type	Participant	Gender
1	Interviews lasted 7–20 mins (mean 12 mins)	Newly-consented ROCSS participants (n = 3)	2 male; 1 female
2	Interviews lasted 7–23 mins (mean 12 mins)	ROCSS participants at a 12-month follow-up appointment (n = 10)	8 male; 2 female

Analysis of results suggested six themes. Themes one to three relate to our first two research questions exploring participants’ use of information sources and the effect this had on their knowledge of the trial. Theme four explores a possible *explanation* for their limited use of this information, as participants provided reasons for their attitudes in a way that was philosophically interesting, although somewhat unexpected. Finally, themes five and six summarise the suggestions that participants had for improving the process.

### Theme one: Varied and limited use of consent discussions

One of the study’s aims was to establish whether and to what extent participants made use of participant information sheets or of information provided in the consent discussions when deciding whether to participate in trials. The newly-consented participants were specifically asked whether they had asked questions in their consent discussions. While follow-up participants were not asked about this specifically, a minority of them talked about the questions they had asked at the time of consent. Across both groups, most of those who commented reported that they did ask questions of the research team (though those who did not ask questions in the follow-up group may have been less likely to bring up the subject):

*I just asked the doctor next door whether I was okay to*, *to go abroad again*… [I’m] *just worried about the insurance part*, *if anything happened*, *or if I was ill*’ (FU5—these codes are the participant identifiers–“FU” signifies “follow-up”, a stage two participant, and “NC” means “newly-consented”, a stage one participant).

One participant said that they wanted to benefit medical science, and therefore did not want to know information about the disadvantages of participating in the trial in case this deterred them from participating:

[I]*f I thought there were downsides*, *I wouldn’t agree*, *because if you’re going to agree to something*, *you need to walk in looking at the positives*, *if you look at the negatives*, *you might not agree to participate*, *so I didn’t give that much attention*, *I didn’t ask* (NC2).

Use of consent discussions was therefore varied, though no participant reported asking more than one question, and two (one NC and one FU) reported asking nothing.

### Theme two: Minimal ongoing use of information sheets

The study also aimed to ascertain the extent to which participants used the information sheets throughout the trial. Only the FU participants were asked directly about this (NC participants were interviewed shortly after their consent discussion, so this was less likely to be applicable to them). Participants’ use of the information sheet over the course of the trial varied. Some reported not having looked at it at all:

*I haven’t looked at it since* (FU6).

[W]*e’d completely forgotten all about* [the trial] (FU7 –referring to himself and his wife).

When participants elaborated on what did or would lead them to revisit their information sheet, they commonly reported that they may revisit information about the trial if they experienced adverse events that they thought were relevant to their condition or surgery. Some of these participants had experienced such adverse events, and had subsequently consulted the trial information:

*I had a wound that’s not healing properly and it said see the doctors but they said it would heal*, *so I was really just that once*, *looking to see*, *for the information* (FU8).

Others had neither experienced such events nor revisited the information:

*I haven’t been back to the information since the procedure*, *mainly because there’s been no issues* (FU10).

Others spoke more abstractly and did not say whether they had experienced such events themselves, but also reported that these events might be key in determining whether they would revisit the trial information:

*I think where it gets important is where there is an incident*, *whatever it is that might be* (FU9).

Overall, the use of information sheets amongst participants as the ROCSS trial went on was minimal. Most FU participants ignored the information sheet unless they experienced health issues that they felt may have been related to the trial or the existing condition to which the trial related.

### Theme three: Awareness of the trial

As the pilot study also aimed to gain an understanding of participants’ awareness of the trial in which they were participating, the topic guide included questions that would require participants to demonstrate at least a basic knowledge of the trial. All three of the NC participants were able to describe the basics of the ROCSS trial:

[T]*hey said that if I consented to do the trial*, *erm*, *it was to something like a mesh*, *so that you*, *if you’re at risk of having a hernia*, *the mesh would potentially prevent that*. *It was a random test*, *and even if you agree to be on the*, *er*, *the trial*, *it doesn’t necessarily mean that you will have had the procedure done* (NC3).

Knowledge amongst the FU participants, however, was highly varied. Some, like the NC participants, were able to describe the basics of the trial, with some being able to describe the different trial arms:

[T]*hey said that my wall would be weakened by what they had to do inside my stomach when they done the operation*, *so by putting this mesh in*, *it was going to like*, *help the strength of it*. *I couldn’t control whether the surgeon put it in or didn’t put it in* (FU3)

One of the participants reported having forgotten about being in the trial until the appointment letter had arrived, and a small number of participants seemed unable to describe *any* element of the trial.

*You see*, *it’d been over a year*, *and maybe a year is too long* [hmm]. *You see*, *I—well we’d–as I say–we’d completely forgotten all about it* (FU7).

*If I had* [the information sheet] *a year ago*, *I probably did scan through it erm*, *but err that’s as much as I can tell you* (FU9).

In summary, while some of the participants had a good knowledge of the basics of the trial, others were very unaware of what the trial was about, or even that they were still part of it.

### Theme four: Participants trusted the medical staff

Many of the participants related their lack of interest in information about the trial to their trust in doctors and medical staff:

*I’m just the guinea pig*. *You do the paperwork and you follow it up and that’s your business*. *I’ll be there for you to practice on*, *or whatever*, *but I’m not interested in paperwork at all* […]. *I’m happy to be the guinea pig*, *but I’m not concerned what comes out of it*. *I’m not gonna go out and get The Lancet and see what studies*, *you know [right]*, *are on trial at the moment*. *So*, *you know*, *I’m willing to help in any capacity*, *but I don’t look at it* (FU2).

*I’m sure as it went on*, *I’d get told all the little details–throughout all the appointments*, *and just going to clinic appointments and checkups and everything* [….] *I just*, *kind of*, *go with what the doctors tell me*, *erm*, *because I’m sure they’re very good at their job and don’t do anything wrong*–(FU1).

FU2’s use of “you” may betray this participant’s mistaken belief that the interviewer for the sub-study was involved in the ROCSS parent trial.

FU1 seemed confident that their doctors would act appropriately, but they also seemed not to appreciate the difference between interventions relating to their treatment and interventions relating to the trial.

In one case, a participant’s trust in the medical team was such that they suggested that the trial be presented to patients as a routine part of the operation, which would circumvent gaining consent:

*I would just say*, *‘you can just—it’s part of the operation* [okay], *part of the routine operation*, *and that’s what we do*, *that’s what we’re gonna do*, *because it’s not gonna harm you*, *but it could benefit you’* […]. *Obviously*, *I wouldn’t override someone that*, *I don’t know*, *you know what I mean*, *he obviously knows more than me* (NC1).

This participant seemed to expect that the research intervention would be beneficial to patients, but it nevertheless provides an interesting (although outlying in terms of this dataset) view about consent being unnecessary. However, this participant thought consent was unnecessary on the basis that participation in the trial would cause them no harm. They appeared not to be aware that there were risks involved in participating (e.g. reduced prophylactic prevention of herniation), even though these were clearly outlined in the PIS.

To summarise this section, many participants seemed to trust that staff would automatically provide them with any information that was important to them personally. They therefore did not feel the need to be proactive about informing themselves, or find it necessary to read the information sheet, which would not provide such personally-tailored information.

### Theme five: Participants wanted more time to read information

Participants’ views about *when* information should be given about trials were also varied. This question was included as a prompt on the topic guide, so participants were asked directly about this. Some of the participants did not receive the written trial information until the day of their surgery, and while it should be noted that this was consistent with the trial’s protocol, some of these participant criticised this timing:

[T]*o be given that information a little bit in advance*, *just gives you that extra kind of breathing room that extra pause to really take the time to think and make a more informed decision for yourself* (FU10).

[I]*f I had had longer to think about it*, *it would have been better* (FU3).

An alternative view, however, voiced by one participant, was that this timing could be a positive thing from the perspective of better ensuring that participants would actually read the information:

[B]*ecause of err*, *that particular environment where you’re locked into it and you are waiting to be called for your operation*, *you’re*, *it’s nice to have something to read because it gives you something to get involved with*, *so yes*, *I think it is good and useful to have in*, *at that point*. *Because if you handed it to me in the street or something*, *I would have gone hmmm*, *and shoved it in my bag and not*, *or like when you get so much information in the post*, *you think oh*, *and you chuck it*, *so it was a different way of receiving but also because you were a captive audience–*(NC2).

In summary, participants tended to emphasise the importance of having sufficient time to read the trial information, with one notable and interesting exception regarding a possible more strategic approach to the timing of information provision.

### Theme six: Participants wanted varied modes of provision

Participants were asked directly about different modes of information provision. They were invited to consider their preferences regarding written paper information, video information on DVDs or in YouTube clips, and an electronic expanding information sheet [15].

A common point raised by the study participants was a concern over newer technologies being used to convey information, as not everyone would have access to them:

*I don’t have a computer or smart phone or such technology (*FU4).

On the other hand, some participants were more enthusiastic about the idea of non-written forms of receiving trial information:

*A DVD for me*. *I don’t do reading* (FU2).

Other participants interpreted the question less subjectively and, acknowledging that different potential participants may wish to receive the information in different ways, suggested that a variety of different modes be made available to participants:

*A little package saying you can get it*, *you can review it this way*, *or you can have a DVD*, *or you can read about it* (NC1).

[P]*eople learn in different ways and take information differently so it’s just best to cover all bases*, *then no one can say that they haven’t been well informed at any stage* (FU10).

To summarise, some participants considered that different modes of receiving information would work better for different people, and individual participants confirmed this by discussing their own preferences.

## Discussion

### Implications for further research

This study has provided valuable pilot data on the consent process for a surgical trial and the use of PIS and consent discussions. Participants reported varying degrees of knowledge about the trial and varying use of the trial information sheet over the course of the trial. Our results also show that there may be some demand for more multi-faceted means of information provision rather than reliance on the information sheet and consent discussions.

The main operational challenge in this study was identifying a suitable trial in which to embed. Working in conjunction with a trial from the outset would streamline the approvals process and provide greater opportunities to sample participants at different stages of the trial.

### Discussion of pilot study results

#### Trial knowledge and use of information and consent discussions

Participants sometimes reported ignorance of the trial, and this gap in the consent process may mean that participants’ autonomy is being compromised, as participants are insufficiently informed for their consent to be valid. Additional measures may therefore be required to ensure that participants are given information that they understand. Further thought may also be needed on the question of how long participants should be expected to retain information, and for how long their continued participation in a trial can be considered autonomous if they begin to forget key details of the trial. Moving to a model of continuous consent, where consent is reaffirmed at stages throughout the trial, would be in line with proposals to use a process-based rather than event-based model of consent [28–29].

Conversely, if participants are unconcerned about trial information yet still happy to participate, a good alternative might be a reframing of the recruitment process to better fit with these desires. If, as indicated in this pilot study, participants’ trust of healthcare practitioners is central to their decision-making, it may be worth exploring the introduction of trust and trustworthiness as operative concepts in place of autonomy in these settings. If research participants are happy to act on the basis that they trust the practitioner who is recruiting them to the study, then perhaps the focus should move towards ensuring that trials are safe and that staff involved with them are acting with integrity, than on providing information to participants who may not really want that information. Such a model would be sympathetic to the proposal, suggested by some authors, to recognise the centrality of trust in the patient-doctor relationship, and move the focus away from individualistic accounts of patient autonomy that overemphasise patient choice and underplay the role of doctors (and the wider medical and research community) in ensuring that patients and research participants make choices that are best for them [30].

Our participants’ recollections of their consent discussions is consistent with previous studies’ findings showing problems with information sheets, includinglimited reading of the PIS, and consequent general limited understanding of the trial itself [10–19]. This may be partly because they assume that the trial will benefit them or others, and that no harm will come to them by participating (the related notion of trusting the doctors and medical staffis discussed in further detail below). The ROCSS trial was not risk-free, but it was a relatively low risk trial. It may be reasonable to suppose that greater potential risks may have prompted participants to pay more attention to the information provided, and more of a reason for researchers to ensure that participants understood this regardless of any apparent lack of interest from them. Further research into participant information in different risk contexts would therefore be useful.

#### Timing of information

Respect for participant autonomy is frequently cited as the reason for ensuring that participants with capacity to do so give informed and voluntary consent. It seems self-evident that good conditions for ensuring autonomous, informed choice are those in which the participant is given *sufficient* time to become acquainted with and consider the trial information. If this is true, it might follow that the more time a participant is given to read the information, the better (within limits–they may forget information if too much time passes). This may provide another reason for some participants’ lack of information-seeking about the trial, as they may not have had sufficient time to formulate questions.

Participant NC2, on the other hand, was glad to receive information on the day of surgery. While not representative of the study population as a whole, this view warrants further attention. It is often thought that being given information close to the time when a decision is required is an obstacle to considered decision-making. This assumption may be worth exploring in future research. A further, larger consultation with patients and the public may help to identify the best time to provide trial information to potential participants, and this timing could then be made explicit in trial protocols and ethics applications. If we wish to maintain a model of recruitment to trials that focuses on autonomy, this may best be served where participants are reminded periodically about their ongoing trial participation. A truly process-based model of consent must take the suggestion of these reminders seriously. These reminders, however, may lead to a higher attrition rate, and further compound the biases introduced by the requirement only to use consenting participants. These are considerations that would need to be weighed against the better preservation of participant autonomy.

#### Trust in medical staff

The participants showed a high level of trust in the medical staff, though in some cases they did not seem able to distinguish between medical staff involved directly in their care and those involved as reseachers in the ROCSS trial. This may have been a product of incorporating trial-related interventions into the usual care pathway as much as possible, for example by arranging ROCSS-related follow-up appointments to take place at normal follow-up clinics that patients would attend irrespective of their participation in ROCSS. It is somewhat paradoxical that a measure designed to minimise the inconvenience to trial participants may actually compromise their knowledge and level of understanding and information about their research participation.

In spite of this confusion, many participants seemed to assume that the trial would be positive in some way and would not disadvantage them. Many suggested that a major factor in their decision to look at the information sheet during the trial would be whether or not they experienced any health issues that could be related to the condition leading them to be in the trial (in this case, the stoma reversal operation). It is therefore perhaps unsurprising that many participants did not look at the trial information–many of them were just happy to think that they were helping. This may also help to explain why some of the follow-up participants were unable to describe even the basic elements of the trial. A similar study in a higher risk trial may yield different results. The participants’ level of trust may also vary from trial to trial depending on the amount of related contact with the staff their condition has required.

Another paradox is illuminated by the participant who argued that potential trial participants do not need information about trials that will not harm them. This participant’s trust in the trial team may have been somewhat misplaced, given that there *were* risks associated with participating in the ROCSS trial, meaning that freedom from harm was not guaranteed. It may have been interesting to probe this further with the participant to verify their awareness of these risks, but asking the participant about these risks may have scared them, or made them feel embarrassed about agreeing to participate without knowing about them. This is therefore an area in which the conduct of the research itself generated ethical issues.

#### Addressing the problem

The fact that participants did not ask questions at the time of enrolment, and that they were sometimes unaware of their participation in clinical trials after a year, suggests either that there is something wrong with the current means by which information is provided to potential trial participants, or that the paradigm that insists that this information is offered is misguided. A dramatic way to resolve this problem would be to acknowledge that potential participants are often uninterested in extensive study information, and accept their trust in the medical profession by allowing them to participate without undergoing the rigorous informed consent process that is insisted on today. Participants would then receive information only on request. Removing this hurdle would allow more participants to realise the social goods of participating in research [30–31].

This trust system could be bolstered by the addition of measures that would make the medical and research community more trust*worthy*, such as greater monitoring of the process of recruitment to trials, for example by having an extra person in the room when participants are enrolled who can ensure that the process is noncoercive.

Stopping short of such a paradigm shift, if we wish to keep using information sheets and recording consent formally, there are two tangible improvements that may address the problem of participants’ poor understanding and retention. The first is through the provision of information using a web-based expanding information sheet, which facilitates reading by introducing information in varying degrees of complexity, and helps the user to select the amount of information they want to read [15]. Second, given the dubiousness of the assumption that a one-off consent process protects participants’ autonomy, a process of refreshing participants’ consent at intervals may better achieve this end [32]. Making alterations to both the mode *and* the timing of information provided to participants may be necessary to avoid the pitfall, encountered by many authors, of information provision becoming an empty “medical Miranda” ritual that does not fulfil its intended function [13, 29, 33].

Our pilot work suggests that online expanding information might be acceptable to potential participants. Some of our participants expressed reluctance to obtain information by reading a conventional information sheet, and others thought that different mediums for providing information could be an advantage. Electronic expanding information provision can accommodate pictures and links to videos in which the trial (or parts of it) is (or are) explained verbally, with pictures, cartoons, other means of communication, or some combination of different media. It also responds to an increasing reliance on and acceptability of electronic forms of communication accessed via mobile devices. We do not expect this to be a panacea, but it may improve the situation by broadening the methods of communication to potential participants, whilst also making the process both more interesting and more convenient.

If those conducting consent discussions are placing more emphasis on the consent discussion over PIS, then ethics committees should consider mirroring this emphasis. This could be achieved quite easily, by putting information into a standardised script that is read to or discussed with all potential participants. This script could also undergo review by the research ethics committee in the same way a PIS does currently.

### Study strengths and limitations

Embedding our pilot into an active trial means that our findings have greater validity than if we had developed hypothetical scenarios for participants to consider. In terms of the number of participants, the time-consuming nature of identifying a suitable and willing trial delayed the project to the extent that we under-recruited–further recruitment might have been possible, but stopped when the project ran out of time, before the point of theoretical saturation. This delay, in combination with the convenience sampling approach necessitated by the conditions under which the trial was undertaken, means that the ‘qualitative power’ [34] of the study (the usefulness of the study’s data relevant to sampling methods and sample size) may be low. A better approach in future would be to approach trial teams while their protocols are still being written, to avoid the need to return to the ethics committee for an amendment, and to allow for a more scientifically robust sampling method. The study nevertheless fulfilled its aims as a pilot study in demonstrating that gathering further data from patients about their use of information, particularly in trials where the risks are different, would be a useful future endeavour.

The ethics committee who reviewed the amendment to the ROCSS protocol recommended that we provided written information sheets and record written consent for our participants. This would not have been required for an audit of the ROCSS consent process. We suspect that this process led to some confusion about the relationship between the ROCSS parent study and our sub-study even though we attempted to mitigate this risk with careful explanation to our participants, both in the information sheets themselves, and in the process of gaining their consent to participate in the sub-study. Nevertheless, if participants were confused about the questions they were being asked in the interviews, this will have affected the study’s results. We were highly conscious that our own study should not deflect attention away from either the patients’ decision to have surgery, or the decision to participate on the ROCSS study. However, work to improve the provision of information to research participants is important and cannot all be conducted hypothetically or with user groups. Consideration needs to be given to how best to gather “real-time” information from participants that is not unduly burdensome, is not a distraction, and provides safeguards that are proportionate to the risks of participation. Data from a pilot of this size should be interpreted with caution, and the passage of time since the data were collected might make some of the participants’ comments out of date (for example, regarding the usefulness of DVD information and greater penetration of the smartphone market). Nevertheless, the study highlights interesting and useful themes.

## Conclusions

**Summary**

- A larger study of a similar nature would be feasible, but embedding the larger study into a trial at a much earlier stage would be more efficient and produce better results.
- Participants reported varying degrees of trial knowledge, and varying use of both consent interviews and trial information over the course of the trial.
- Participants placed a high degree of trust in medical staff. It may be worth exploring the introduction of trust as an operative concept in place of autonomy in these settings. Further research to establish any factors affecting this level of trust would be useful.
- Future potential trial participants may benefit from different methods of receiving information, including different approaches to timing.

Our pilot study corroborates previous work suggesting that trial participants’ use of different means of information provision is highly varied, with some participants reporting having asked no questions in the consent discussion, and some reporting limited or no use of the paper information sheet provided to them. Likely as a consequence of this, participants were sometimes unaware of the nature of trials in which they were participating, or even whether they were participating at all. Our findings suggest that alternative methods, including non-written methods of trial information provision may be at least as acceptable, and may improve participants’ understanding of trials, protecting their autonomy with regard to making potentially risky decisions.

## Supporting information

S1 Appendix Topic guide. Interviewer’s prompt sheet.
(DOCX)

## References

[1] Beauchamp T, Childress J. Principles of biomedical ethics. 7^th^ ed USA: Oxford University Press; 2013.

[2] Luna F, Macklin R. Research involving human beings In: Kuhse H, Singer P, editors. A companion to bioethics. Chichester: Wiley-Blackwell; 2009 p. 457–468.

[3] Doyal L. Journals should not publish research to which patients have not given fully informed consent–with three exceptions. BMJ. 1997;314:1107–11. 10.1136/bmj.314.7087.1107 9133897 PMC2126464

[4] Faden R, Beauchamp T. A history and theory of informed consent. New York: Oxford University Press; 1986.

[5] O'Neill O. Some limits of informed consent. J Med Ethics. 2003;29:4–7. 10.1136/jme.29.1.4 12569185 PMC1733683

[6] The Nuremberg code (1947). Br Med J. 1996;313:1448.

[7] World Medical Association. Declaration of Helsinki—ethical principles for medical research involving human subjects. 2013. https://www.wma.net/policies-post/wma-declaration-of-helsinki-ethical-principles-for-medical-research-involving-human-subjects/ Accessed 18 Jan 2018. 10.1001/jama.2013.281053 24141714

[8] European Commission. Clinical trials directive (2001/20/EC). 2001. http://ec.europa.eu/health/files/eudralex/vol-1/dir_2001_20/dir_2001_20_en.pdf. Accessed 18 Jan 2018.

[9] The medicines for human use (clinical trials) regulations 2004. http://www.legislation.gov.uk/uksi/2004/1031/pdfs/uksi_20041031_en.pdf. Accessed 1 Jan 2018. 15812991

[10] Davis T, Holcombe R, Berkel H, Pramanik S, Divers S. Informed consent for clinical trials: a comparative study of standard versus simplified forms. J Natl Cancer Inst. 1998;90:668–74. 10.1093/jnci/90.9.668 9586663

[11] Antoniou E, Draper H, Reed K, Burls A, Southwood T, Zeegers M. An empirical study on the preferred size of the participant information sheet in research. J Med Ethics; 2011;37:559–62. 10.1136/jme.2010.041871 21478421

[12] Joffe S, Cook E, Cleary P, Clark J, Weeks J. Quality of informed consent in cancer clinical trials: a cross-sectional survey. Lancet. 2001;9295:1772–7. 10.1016/S0140-6736(01)06805-2 11734235

[13] Dixon-Woods M, Ashcroft R, Jackson C, Tobin M, Kivits J, Burton P, et al Beyond “misunderstanding”: written information and decisions about taking part in a genetic epidemiology study. Soc Sci Med. 2007;11:2212–22. 10.1016/j.socscimed.2007.08.010 17904716

[14] Ennis L, Wykes T. Sense and readability: Participant information sheets for research studies. Br J Psychiatry. 2016;208:189–94. 10.1192/bjp.bp.114.156687 26382948 PMC4837385

[15] Kirkby H, Calvert M, McManus R, Draper H. Informing potential participants about research: observational study with an embedded randomized control trial. PLoS One. 2013;8:e76435 10.1371/journal.pone.0076435 24098499 PMC3789820

[16] Jefford M, Moore R. Improvement of informed consent and the quality of consent documents. Lancet Oncol. 2008;9:485–93. 10.1016/S1470-2045(08)70128-1 18452859

[17] Brehaut J, Carroll K, Elwyn G, Saginur R, Kimmelman J, Shojania K, et al Informed consent documents do not encourage good-quality decision making. J Clin Epidemiol. 2012;65:708–24. 10.1016/j.jclinepi.2012.01.004 22537428

[18] Falagas M, Korbila I, Giannopoulou K, Kondilis B, Peppas G. Informed consent: how much and what do patients understand?. Am J Surg. 2009;198:420–35. 10.1016/j.amjsurg.2009.02.010 19716887

[19] Kiguba R, Kutyabami P, Kiwuwa S, Katabira E, Sewankambo N. Assessing the quality of informed consent in a resource-limited setting: a cross-sectional study. BMC Med Ethics. 2012;13:21 10.1186/1472-6939-13-21 22906301 PMC3478970

[20] Medical Research Council. Guidelines for good clinical practice in clinical trials. 1998. http://mrc.ac.uk/documents/pdf/good-clinical-practice-in-clinical-trials. Accessed 18 Jan 2018.

[21] Reinforcement of Closure of Stoma Site (ROCSS) Collaborative and West Midlands Research Collaborative. Prophylactic biological mesh reinforcement versus standard closure of stoma site (ROCSS): a multicentre, randomised controlled trial. Lancet. 2020;395:417–26. 10.1016/S0140-6736(19)32637-6 32035551 PMC7016509

[22] Bryman A. Social research methods. 4th ed Oxford: Oxford University Press; 2012 10.1016/j.ymeth.2012.10.003

[23] Taylor MC. Interviewing In: Holloway I, editor. Qualitative research in health care. New York: Open University Press; 2005;39–55.

[24] Kartch F. Narrative interviewing In: The SAGE encyclopedia of communication research methods. Thousand Oaks: SAGE Publications; 2017.

[25] Chase SE. Narrative inquiry: toward theoretical and methodological maturity In: The SAGE handbook of qualitative research. Thousand Oaks: SAGE Publications; 2018.

[26] Corbin J, Strauss A. Basics of qualitative research: techniques and procedures for developing grounded theory. 3rd ed London: SAGE Publications; 2008.

[27] Silverman D. Doing qualitative research. 4th ed Los Angeles: SAGE Publications; 2013.

[28] Lidz C, Appelbaum P, Meisel A. Two models of implementing informed consent. Arch Intern Med. 1988;148:1385–9. 3377623

[29] Ali V. Consent forms as part of the informed consent process: moving away from medical Miranda. Hastings Law J. 2003;54:1575–92. 15199939

[30] Harris J. Scientific research is a moral duty. J Med Ethics. 2005;31:242–8. 10.1136/jme.2005.011973 15800367 PMC1734128

[31] Chalmers I. Regulation of therapeutic research is compromising the interests of patients. Pharmaceut Med. 2007;21:395–404.

[32] Cox K. Informed consent and decision-making: patients’ experiences of the process of recruitment to phases I and II anti-cancer drug trials. Patient Educ Couns. 2002;46:31–8. 10.1016/s0738-3991(01)00147-1 11804767

[33] Armstrong N, Dixon-Woods M, Thomas A, Rusk G, Tarrant C. Do informed consent documents for cancer trials do what they should? A study of manifest and latent functions. Sociol Health Illn. 2012;34:1230–45. 10.1111/j.1467-9566.2012.01469.x 22443456

[34] Onwuegbuzie A, Leech N. A call for qualitative power analyses. Qual Quant. 2007;41:105–21.

