# Supplementary material for: Potential research participants’ use of information during the consent process: A qualitative pilot study of patients enrolled in a clinical trial
Source: PLoS One. 2020 Jun 18;15(6):e0234388. doi: 10.1371/journal.pone.0234388 (PMC7302495; doi:10.1371/journal.pone.0234388)
Supplement: S1 Appendix — Interviewer’s prompt sheet. (DOCX) [file pone.0234388.s001.docx]

**Stage 1 interviews**

Thank you for agreeing to talk to me. I want to talk to you about the appointment you had with [name of doctor/researcher gaining consent] when (s)he invited you to participate in the ROCSS study. I only want to talk to you about the part of that appointment when you discussed taking part in the research [name of doctor/researcher gaining consent] is conducting. You do not have to tell me anything private about your condition or treatment.

1. When [name of doctor/researcher gaining consent] was telling you about the research, which piece of information did you find most striking?

Prompts

- Can you remember anything at all about the trial (not the whole operation but the bit that was part of the trial – patient may recall whole operation but not which part ROCSS is about so may need to probe without providing information)
- Is there anything about the trial that sticks out in your mind?

1. What do you think the good things about participating in this clinical trial will be?

Prompts

- Why did you think you would like to take part?

1. What, if anything, did you think might be a downside to taking part?

Prompts

- Was there anything about the trial that concerned you about taking part?

1. What questions did you ask, if any, about the research, in your appointment?
2. Which piece of information about the trial most influenced your decision to take part?

Prompts

- Was this something you read about (if so where?) or something that you discussed in your appointment with [name of doctor/researcher gaining consent]?

We are thinking about different ways of providing information about clinical trials. One suggestion is replacing the written information like this (refer to sheet) with a DVD and/or YouTube type clip on the internet. Alternatively, the information could be presented using an application for a smartphone.

1. What do you think of this idea?

Prompts

- Explore whether they would be more/as likely to watch a DVD or YouTube video than read an information sheet
- Explore whether there is something reassuring about written information – e.g. as something that can be looked at again and whether the same could be true for non-written information
- Would you want written information and the non-written information? Why? What would you use each for?
- When do you think people would read/access information about the research – before/during/after appointment at hospital where first discussed?

**Stage 2 interviews**

Thank you for agreeing to talk to me. There are no right or wrong answers to any of my questions – this isn’t a test of you or any of the doctors and nurses that have looked after you. [Time period] ago you kindly agree to take part in a clinical trial called ROCSS. This research was part of the surgery you had to close your stoma.

1. Where do you keep the information sheet you were given about this study?

Prompts

- I am only interested in the special information that you were given about the trial – the sheet looked like this (show the patient a ROCSS information sheet)
- Where did you keep any of the information that you were given about the surgery or coming into the hospital for the surgery?

1. How often did/do you look at the information sheet for this study?

Prompts

- It doesn’t matter if you haven’t looked at it at all
- How much do you look at any of the written information you are given by the hospital or the staff looking after you – that is not just the information about ROCSS?

1. If you have used it, or currently use it to look for particular information, what kind of information did/do you look for?
2. What kind of information do you think people might want to go back and look at again when they take part in clinical research?

We are thinking about different ways of providing information about clinical trials. One suggestion is replacing the written information like this (refer to sheet) with a DVD and/or YouTube type clip on the internet. Alternatively, the information could be presented using an application for a smartphone.

1. What do you think of this idea?

Prompts

- Explore whether they would be more/as likely to watch a DVD or YouTube video than read an information sheet
- Explore whether there is something reassuring about written information – e.g. as something that can be looked at again and whether the same could be true for non-written information
- Would you want written information and the non-written information? Why? What would you use each for?
- When do you think people would read/access information about the research – before/during/after appointment at hospital where first discussed?
